# Family History and Solar Insolation in Bipolar I Disorder

**DOI:** 10.1111/acps.70075

**Published:** 2026-02-24

**Authors:** M. Bauer, T. Glenn, E. D. Achtyes, M. Alda, E. Agaoglu, K. Altınbaş, O. A. Andreassen, E. Angelopoulos, R. Ardau, M. Aydin, Y. Ayhan, C. Baethge, R. Bauer, B. T. Baune, C. Balaban, C. Becerra‐Palars, A. P. Behere, H. Belete, T. Belete, G. Okawa Belizario, F. Bellivier, R. H. Belmaker, F. Benedetti, M. Berk, Y. Bersudsky, Ş. Bicakci, H. Birabwa‐Oketcho, T. D. Bjella, C. Brady, J. Cabrera, M. Cappucciati, A. M. Paredes Castro, W. Chen, E. Y. W. Cheung, S. Chiesa, M. Chanopoulou, M. Crowe, A. Cuomo, S. Dallaspezia, P. Desai, S. Dodd, B. Etain, A. Fagiolini, F. T. Fellendorf, E. Ferensztajn‐Rochowiak, J. G. Fiedorowicz, K. N. Fountoulakis, M. A. Frye, P. A. Geoffroy, M. J. Gitlin, A. Gonzalez‐Pinto, J. F. Gottlieb, P. Grof, B. C. M. Haarman, H. Harima, M. Hasse‐Sousa, C. Henry, L. Hoffding, J. Houenou, M. Imbesi, E. T. Isometsä, M. Ivkovic, S. Janno, S. Johnsen, F. Kapczinski, G. N. Karakatsoulis, M. Kardell, L. V. Kessing, S. J. Kim, B. König, T. L. Kot, M. Koval, M. Kunz, B. Lafer, M. Landén, E. R. Larsen, R. W. Licht, V. M. Ludwig, C. Lopez‐Jaramillo, A. MacKenzie, H. Østergaard Madsen, S. Alberte Kongstad A. Madsen, J. Mahadevan, A. Mahardika, K. Mahfoudh, M. Manchia, W. Marsh, M. Martinez‐Cengotitabengoa, J. Martini, K. Martiny, Y. Mashima, D. M. McLoughlin, A. N. R. Meesters, Y. Meesters, I. Melle, F. Meza‐Urzúa, E. Michaelis, P. Mikolas, Y. Ming Mok, S. Monteith, M. Moorthy, G. Morken, E. Mosca, A. A. Mozzhegorov, R. Munoz, S. V. Mythri, R. K. Nadella, T. Nakanotani, R. Ernst Nielsen, C. O'Donovan, A. Omrani, Y. Osher, U. Ouali, M. Pantovic‐Stefanovic, P. Pariwatcharakul, J. Petite, A. Pfennig, M. Pilhatsch, Y. Pica Ruiz, M. Pinna, M. Pompili, R. Porter, D. Quiroz, F. Diego Rabelo‐da‐Ponte, R. Ramesar, N. Rasgon, W. Ratta‐apha, M. Redahan, M. S. Reddy, A. Reif, E. Z. Reininghaus, J. Gringer Richards, P. Ritter, J. K. Rybakowski, L. Sathyaputri, A. M. Scippa, C. Simhandl, D. Smith, J. Smith, P. W. Stackhouse, D. J. Stein, K. Stilwell, S. Strejilevich, K.‐P. Su, M. Subramaniam, A. Hatim Sulaiman, K. Suominen, A. J. Tanra, Y. Tatebayashi, W. Lin Teh, L. Tondo, C. Torrent, D. Tuinstra, T. Uchida, A. E. Vaaler, E. Vieta, B. Viswanath, C. Volf, K.‐J. Yang, M. Yoldi‐Negrete, O. Kaan Yalcinkaya, A. H. Young, Y. Zgueb, P. C. Whybrow

**Affiliations:** ^1^ Department of Psychiatry and Psychotherapy, University Hospital Carl Gustav Carus, Faculty of Medicine Technical University Dresden Dresden Germany; ^2^ ChronoRecord Association Fullerton California USA; ^3^ Department of Psychiatry Western Michigan University Homer Stryker M.D. School of Medicine Kalamazoo Michigan USA; ^4^ Department of Psychiatry Dalhousie University Halifax Nova Scotia Canada; ^5^ Department of Psychiatry Hacettepe University Faculty of Medicine Ankara Turkey; ^6^ Department of Psychiatry Atlas University Istanbul Turkey; ^7^ Division of Mental Health and Addiction, Oslo University Hospital & Institute of Clinical Medicine University of Oslo Oslo Norway; ^8^ Department of Psychiatry National and Capodistrian University of Athens, Medical School, Eginition Hospital Athens Greece; ^9^ Section of Neurosciences and Clinical Pharmacology, Department of Biomedical Sciences University of Cagliari Sardinia Italy; ^10^ Department of Psychiatry Selcuk University Faculty of Medicine Konya Turkey; ^11^ Department of Psychiatry and Psychotherapy, Faculty of Medicine University of Cologne Cologne Germany; ^12^ Department of Psychiatry University of Münster Münster Germany; ^13^ Department of Psychiatry, Melbourne Medical School The University of Melbourne Melbourne Australia; ^14^ The Florey Institute of Neuroscience and Mental Health The University of Melbourne Parkville Victoria Australia; ^15^ University Hospital, Department of Psychiatry, Psychosomatic Medicine and Psychotherapy Goethe University Frankfurt Frankfurt Germany; ^16^ National Institute of Psychiatry “Ramón de la Fuente Muñiz” Mexico City Mexico; ^17^ Department of Pediatrics and Human Development Michigan State University Grand Rapids Michigan USA; ^18^ Department of Psychiatry, College of Medicine and Health Sciences Bahir Dar University Bahir Dar Ethiopia; ^19^ Bipolar Disorder Research Program, Department of Psychiatry University of São Paulo Medical School São Paulo Brazil; ^20^ Département de Psychiatrie et de Médecine Addictologique, Assistance Publique – Hôpitaux de Paris, INSERM UMR‐S1144 Université Paris‐Cité, FondaMental Foundation Paris France; ^21^ Professor Emeritus of Psychiatry Ben Gurion University of the Negev Beer Sheva Israel; ^22^ University Vita‐Salute San Raffaele Milan Italy; ^23^ Irccs Ospedale San Raffaele Milano Italy; ^24^ IMPACT – The Institute for Mental and Physical Health and Clinical Translation, School of Medicine, Barwon Health Deakin University Geelong Australia; ^25^ Orygen, The National Centre of Excellence in Youth Mental Health, Centre for Youth Mental Health, Florey Institute for Neuroscience and Mental Health and the Department of Psychiatry The University of Melbourne Melbourne Australia; ^26^ Department of Psychiatry, Faculty of Health Sciences, Beer Sheva Mental Health Center Ben Gurion University of the Negev Beer Sheva Israel; ^27^ Department of Psychiatry Baskent University Faculty of Medicine Ankara Turkey; ^28^ Butabika Hospital Kampala Uganda; ^29^ Department of Psychiatry, Trinity College Dublin St Patrick's University Hospital Dublin Ireland; ^30^ Mood Disorders Clinic Dr. Jose Horwitz Psychiatric Institute Santiago de Chile Chile; ^31^ Department of Mental Health and Substance Abuse Piacenza Italy; ^32^ Department of Psychiatry, Chiayi Branch Taichung Veterans General Hospital Chiayi Taiwan; ^33^ Private Practice Central, Hong Kong China; ^34^ Department of Psychiatry, School of Medicine, Faculty of Health Sciences Aristotle University of Thessaloniki Thessaloniki Greece; ^35^ Department of Psychological Medicine University of Otago Christchurch New Zealand; ^36^ Department of Molecular Medicine University of Siena School of Medicine Siena Italy; ^37^ Pine Rest Christian Mental Health Services Grand Rapids Michigan USA; ^38^ Department of Psychiatry University of Melbourne Parkville Victoria Australia; ^39^ Division of Psychiatry and Psychotherapeutic Medicine Medical University Graz Graz Austria; ^40^ Department of Adult Psychiatry Poznan University of Medical Sciences Poznan Poland; ^41^ The Ottawa Hospital and University of Ottawa Ottawa Ontario Canada; ^42^ Department of Psychiatry & Psychology Mayo Clinic Depression Center, Mayo Clinic Rochester Minnesota USA; ^43^ Département de psychiatrie et d'addictologie, AP‐HP, GHU Paris Nord, DMU Neurosciences Hopital Bichat ‐ Claude Bernard Paris France; ^44^ Centre ChronoS GHU Paris ‐ Psychiatry & Neurosciences Paris France; ^45^ NeuroDiderot, Inserm Université de Paris Paris France; ^46^ Department of Psychiatry, GHU Paris Psychiatrie & Neurosciences Université de Paris Paris France; ^47^ Department of Psychiatry and Biobehavioral Sciences, Semel Institute for Neuroscience and Human Behavior University of California Los Angeles (UCLA) Los Angeles California USA; ^48^ BIOARABA. Department of Psychiatry, University Hospital of Alava University of the Basque Country, CIBERSAM Vitoria Spain; ^49^ Department of Psychiatry, Feinberg School of Medicine Northwestern University Chicago Illinois USA; ^50^ Mood Disorders Center of Ottawa and the Department of Psychiatry University of Toronto Canada; ^51^ Department of Psychiatry, University Medical Center Groningen University of Groningen Groningen the Netherlands; ^52^ Department of Psychiatry Tokyo Metropolitan Matsuzawa Hospital Setagaya Tokyo Japan; ^53^ Department of Psychiatry Universidade Federal do Rio Grande do Sul Porto Alegre Brazil; ^54^ Programa de Pós‐Graduação em Psicologia, Departamento de Psicologia do Desenvolvimento e da Personalidade, Instituto de Psicologia Universidade Federal do Rio Grande do Sul Porto Alegre Brazil; ^55^ Department of Clinical Research University of Southern Denmark Odense Denmark; ^56^ INSERM, IMRB, Translational Neuropsychiatry, APHP, Mondor Univ Hospitals, Fondation FondaMental Université Paris Est Créteil Créteil France; ^57^ Université Paris Saclay, CEA, Neurospin Gif‐sur‐Yvette France; ^58^ Department of Psychiatry University of Helsinki and Helsinki University Hospital Helsinki Finland; ^59^ National Institute for Health and Welfare Helsinki Finland; ^60^ University Clinical Center of Serbia, Clinic for Psychiatry Belgrade Serbia; ^61^ Department of Psychiatry University of Tartu Tartu Estonia; ^62^ Unit for Psychiatric Research Aalborg University Hospital Aalborg Denmark; ^63^ Department of Psychiatry and Neurochemistry, Institute of Neuroscience and Physiology, The Sahlgrenska Academy University of Gothenburg Gothenburg Sweden; ^64^ Copenhagen Affective Disorder Research Center (CADIC), Psychiatric Center Copenhagen Copenhagen Denmark; ^65^ Department of Psychiatry Chosun University School of Medicine Gwangju Republic of Korea; ^66^ BIPOLAR Zentrum Wiener Neustadt Wiener Neustadt Austria; ^67^ Khanty‐Mansiysk Clinical Psychoneurological Hospital Khanty‐Mansiysk Russia; ^68^ Department of Neuroscience Michigan State University East Lansing Michigan USA; ^69^ Department of Medical Epidemiology and Biostatistics, Karolinska Institutet Stockholm Sweden; ^70^ Mental Health Department Odense, University Clinic and Department of Regional Health Research University of Southern Denmark Esbjerg Denmark; ^71^ Psychiatry – Aalborg University Hospital Aalborg Denmark; ^72^ Department of Clinical Medicine Aalborg University Aalborg Denmark; ^73^ Mood Disorders Program, Hospital Universitario San Vicente Fundación, Research Group in Psychiatry, Department of Psychiatry, Faculty of Medicine Universidad de Antioquia Medellín Colombia; ^74^ Forensic Psychiatry University of Glasgow, NHS Greater Glasgow and Clyde Glasgow UK; ^75^ Copenhagen University Hospitals, Psychiatric Centre Copenhagen Copenhagen Denmark; ^76^ Department of Psychiatry National Institute of Mental Health and Neuro Sciences (NIMHANS) Bengaluru India; ^77^ Department of Psychiatry, Faculty of Medicine Mataram University Mataram Indonesia; ^78^ Razi Hospital, Faculty of Medicine University of Tunis‐El Manar Tunis Tunisia; ^79^ Department of Pharmacology Dalhousie University Halifax Nova Scotia Canada; ^80^ Section of Psychiatry, Department of Medical Science and Public Health University of Cagliari Cagliari Italy; ^81^ Unit of Clinical Psychiatry University Hospital Agency of Cagliari Cagliari Italy; ^82^ Department of Psychiatry University of Massachusetts Medical School Worcester Massachusetts USA; ^83^ School of Pharmacy, University of the Basque Country (UPV/EHU). Basque Sustainable Pharmacy and Biotherapy Research Group 01006 Vitoria‐Gasteiz Spain; ^84^ Osakidetza‐ Basque Health Service, BioAraba Health Research Institute 01006 Vitoria‐Gasteiz Spain; ^85^ Faculty of Health Sciences, International University of La Rioja (UNIR), Neurohealth Research Group 26006 Logroño Spain; ^86^ Department of Neuropsychiatry Keio University School of Medicine Tokyo Japan; ^87^ Dept of Psychiatry & Trinity College Institute of Neuroscience Trinity College Dublin, St Patrick's University Hospital Dublin Ireland; ^88^ Department of Child and Adolescent Psychiatry and Psychotherapy, SHG Klinikum Idar‐Oberstein Germany; ^89^ Department of Mood and Anxiety Disorders Institute of Mental Health Singapore Singapore; ^90^ Michigan State University College of Human Medicine, Traverse City Campus Traverse City Michigan USA; ^91^ Department of Mental Health St Olav University Hospital Trondheim Norway; ^92^ Faculty of Medicine and Health Sciences Norwegian University of Science and Technology – NTNU Trondheim Norway; ^93^ Soviet Psychoneurological Hospital Urai Russia; ^94^ Department of Psychiatry University of California San Diego San Diego California USA; ^95^ Makunda Christian Leprosy and General Hospital Bazaricherra Assam India; ^96^ Department of Psychiatry, NorthWest Area Mental Health Service Northern Hospital Melbourne Australia; ^97^ Affective Disorders Research Project Tokyo Metropolitan Institute of Medical Science Setagaya Tokyo Japan; ^98^ Tunisian Bipolar Forum Tunis Tunisia; ^99^ Department of Psychiatry, Faculty of Medicine Siriraj Hospital Mahidol University Bangkok Thailand; ^100^ Hospital “Ángeles del Pedregal” Mexico City Mexico; ^101^ Lucio Bini Mood Disorder Center Cagliari Italy; ^102^ Department of Neurosciences, Mental Health and Sensory Organs, Sant'Andrea Hospital Sapienza University of Rome Rome Italy; ^103^ School of Medicine Universidad Diego Portales CL Santiago de Chile Chile; ^104^ School of Pharmacy and Biomedical Sciences University of Central Lancashire Preston Lancashire UK; ^105^ SA MRC Genomic and Precision Medicine Research Unit, Division of Human Genetics, Department of Pathology, Institute of Infectious Diseases and Molecular Medicine University of Cape Town South Africa; ^106^ Department of Psychiatry and Behavioral Sciences Stanford School of Medicine Palo Alto California USA; ^107^ Asha Bipolar Clinic Asha Hospital Hyderabad Telangana India; ^108^ Department of Psychiatry, Epidemiology, and Internal Medicine, Iowa Neuroscience Institute The University of Iowa Iowa City Iowa USA; ^109^ Department of Neuroscience and Mental Health Federal University of Bahia Salvador Brazil; ^110^ Bipolar Zentrum Wiener Neustadt Sigmund Freud Privat Universität Vienna Austria; ^111^ Centre for Clinical Brain Sciences University of Edinburgh Edinburgh, Scotland UK; ^112^ AREA, Assistance and Research in Affective Disorders Buenos Aires Argentina; ^113^ Science Directorate/Climate Science Branch National Aeronautics and Space Administration (NASA) Langley Research Center Hampton Virginia USA; ^114^ SAMRC Unit on Risk & Resilience in Mental Disorders, Dept of Psychiatry & Neuroscience Institute University of Cape Town Cape Town South Africa; ^115^ College of Medicine China Medical University (CMU) Taichung Taiwan; ^116^ An‐Nan Hospital China Medical University Tainan Taiwan; ^117^ Research Division Institute of Mental Health Singapore; ^118^ Department of Psychological Medicine, Faculty of Medicine University of Malaya Kuala Lumpur Malaysia; ^119^ Mutual Insurance Company Ilmarinen Helsinki Finland; ^120^ Department of Psychiatry, Faculty of Medicine Hasanuddin University Makassar Indonesia; ^121^ McLean Hospital‐Harvard Medical School Boston Massachusetts USA; ^122^ Mood Disorder Lucio Bini Centers Cagliari Italy; ^123^ Bipolar and Depressive Disorders Unit, Hospital Clinic de Barcelona Fundació de Recerca Clínic Barcelona‐Institut d'Investigacions Biomèdiques August Pi i Sunyer (IDIBAPS) Barcelona Spain; ^124^ Bipolar and Depressive Disorders Unit, Hospital Clinic de Barcelona, Institute of Neurosciences (UBNeuro) Centro de Investigación Biomédica en Red de Salud Mental (CIBERSAM) Barcelona Spain; ^125^ Melbourne Neuropsychiatry Centre, Department of Psychiatry The University of Melbourne Melbourne Australia; ^126^ Subdirección de Investigaciones Clínicas. Instituto Nacional de Psiquiatría Ramón de la Fuente Muñíz Mexico City Mexico; ^127^ Department of Psychological Medicine, Institute of Psychiatry Psychology and Neuroscience, King's College London London UK

## Abstract

**Background:**

Sunlight has profound impacts on physical and mental health, beyond vision, including effects on circadian rhythms, alertness, mood, and sleep. A family history of any mood disorders is strongly associated with psychiatric disorders including bipolar disorder. The purpose of this study was to evaluate the association between a family history of any mood disorder in patients with bipolar I disorder and solar insolation at varied international onset locations.

**Methods:**

Data for this analysis were available from 5842 patients with a diagnosis of bipolar I disorder obtained at 83 collection sites in both hemispheres. This included 4752 patients from 71 collection sites in the northern hemisphere and 1090 patients from 12 collection sites in the southern hemisphere. Patient data variables were obtained from records or interviews. Solar insolation data were obtained from The National Aeronautics and Space Administration (NASA) Power database for each onset location, and the ratio of the mean monthly minimum/mean monthly maximum solar insolation was calculated. Typically, the ratio is largest near the equator (little yearly change in solar insolation) and smallest near the poles (large yearly change in solar insolation).

**Results:**

A significant relationship was found between a family history of any mood disorder, the ratio of the mean monthly minimum/mean monthly maximum solar insolation, and gender. The odds of a family history of mood disorder increased as patient location nears the poles and decreased near the equator. Female gender also increased the odds of having a family history of a mood disorder.

**Conclusions:**

This study highlighted the association between family history, solar insolation, and gender in international patients with bipolar I disorder. Given the profound effects of sunlight on human health, the family of patients with bipolar disorder who live in the same location with the same solar insolation, and especially females, may be at increased risk for a mood disorder.

## Summary

- Significant outcomes
  ○ There is a significant association between family history of a mood disorder, solar insolation, and gender in international patients with bipolar I disorder.
  ○ The odds of a family history of a mood disorder increase as patient location nears the poles and decrease near the equator.
  ○ Family members who live in the same location and experience the same solar insolation, especially females, may be at increased risk for a mood disorder.
- Limitations
  ○ Family history was obtained from patient data. Family members were not interviewed.
  ○ Unique patient light exposure data was not obtained.
  ○ Individual patient sensitivity to light was not analyzed.

## 1 Introduction

Sunlight has a profound, diverse influence on human physiology and health that extends beyond vision, including effects on circadian rhythms, mood, alertness, and sleep [1, 2, 3]. The pattern of solar insolation across the earth's surface varies with the annual changes in the relationship between the earth and sun, and differs by latitude [4]. Using sunlight measured as solar insolation (incoming solar radiation on the surface of the earth), we previously investigated the effects of changes in solar insolation using a large, international database of patients with bipolar I disorder (BD I) from multiple continents and found that the maximum monthly increase in solar insolation was inversely related to the age of onset of BD I [5, 6].

Sunlight is the strongest signal used to synchronize human circadian clocks to the 24 h rotation of the Earth [7, 8]. Nearly all human physiological processes include circadian timing cycles, with a master pacemaker in the suprachiasmatic nucleus and organized peripheral cells containing clock components to produce circadian rhythms [9, 10]. In addition to rods and cones, melanopsin is a special photopigment in the eye that responds to light in the environment [11]. Disruptions in circadian rhythms are associated with a variety of acute and chronic health problems, both physical and mental, including sleep and mood disturbances in bipolar disorder [1, 12, 13, 14]. Additionally, sunlight exposure is the primary source of Vitamin D for both adults and children [15]. The purpose of this analysis was to examine and clarify the relationship between a family history of any mood disorder for patients with BD I and solar insolation at varied onset locations.

## 2 Aim of the Study

Given the importance of sunlight on human physical and mental health, the aim of this study was to investigate the relationship between solar insolation and a family history of any mood disorder in patients with BD 1, using a large, international database with locations extending from the poles to the equator.

## 3 Methods

### 3.1 Patient Data Collection

All patients in the study received a diagnosis of BD I from a psychiatrist according to DSM‐IV, DSM‐5, or ICD criteria. Data were obtained by direct questioning, record review, or both, from individual practitioners, medical centers, and specialty clinics. Approval from local institutional review boards was obtained according to local requirements. The data collected for each patient included diagnosis, gender, age of onset, date of birth, polarity of first episode, family history of mood disorders (other than the patient), history of psychosis, episode course, history of alcohol or substance abuse, history of suicide attempts, birth location, onset location, and current location. Data were collected from 8657 patients with BD I disorder. Patients were excluded if they were missing family history, gender, or if they had different birth and onset locations, leaving 5842 patients with BD I for the analysis. Of the 5842 patients, 4752 were from the northern hemisphere and 1090 from the southern hemisphere.

Data were obtained from 83 collection sites with 71 locations in the northern hemisphere and 12 in the southern hemisphere. In the northern hemisphere, data collection sites included: Austria: Graz, Wiener Neustadt; Canada: Calgary, Halifax, Ottawa; China: Hong Kong; Colombia: Medellín; Denmark: Aalborg, Aarhus, Copenhagen, Odense; Ethiopia: Barhir Dar; Estonia: Tartu; Finland: Helsinki; France: Paris (2 sites); Germany: Dresden, Frankfurt, Würzburg; Greece: Athens, Thessaloniki (2 sites); India: Bengaluru, Hyderabad, Wardha; Ireland: Dublin; Israel: Beer Sheva; Italy: Cagliari, Sardinia (2 sites), Milan, Piacenza, Rome, Siena; Japan: Chiba, Tokyo (3 sites); Malaysia: Kuala Lumpur; Mexico: Mexico City; Netherlands: Groningen; Norway: Oslo, Trondheim; Poland: Poznan; Russia: Khanti‐Mansiysk; Serbia: Belgrade; Singapore; South Korea: Jincheon; Spain: Barcelona, Vitoria; Sweden: Gothenburg, Stockholm; Taiwan: Taichung; Thailand: Bangkok; Turkey: Ankara, Konya; Tunisia: Tunis; Uganda: Kampala; UK: Glasgow; and USA: Grand Rapids, MI, Iowa City, IA, Kansas City, KS, Los Angeles, CA, Palo Alto, CA, Rochester, MN, and San Diego, CA. In the southern hemisphere, data collection sites included: Australia: Adelaide; Argentina: Buenos Aires; Brazil: Porto Alegre, Salvador, São Paulo; Chile: Santiago (2 sites); Indonesia: Mataram; New Zealand: Christchurch; and South Africa: Cape Town.

### 3.2 Statistics

The generalized estimating equations (GEE) statistical technique was used to account for both the correlated data and unbalanced number of patients at collection sites. The GEE technique estimates the dependent variable as a function of the entire population, rather than within a cluster, producing a population averaged or marginal estimate of model coefficients [16]. All GEE models were estimated using a binomial distribution, an exchangeable working correlation matrix, and a logit link function where family history of mood disorders was the dependent binary variable. Confidence intervals at the 0.01 significance level were used to reduce the chance of type 1 errors. Demographic variables were reported using descriptive statistics. SPSS version 30.0 was used for all analyses.

Because the patient birth and onset locations were the same, the insolation values at the patient onset location were used as a proxy for other family members to evaluate the family history. While this assumption may be imperfect, it provides an approximate estimate of insolation for family members.

### 3.3 Solar Insolation

The solar insolation data were obtained from The National Aeronautics and Space Administration (NASA) Power database [4]. Solar insolation refers to the amount of electromagnetic energy from the sun received on earth for a given surface area at a given time, expressed as kilowatt hour/square meter/day (kWh/m^2^/day). The yearly pattern of solar insolation varies by latitude, with few monthly changes at the equator and large changes near the poles. Various other factors also influence solar insolation values, including time of day, altitude, season, local weather, and atmospheric conditions such as air pollution [4, 17]. Rather than a winter summer pattern, tropical locations (less than 23.5° latitude) may have a wet season where clouds decrease insolation and a dry season with clear skies.

To accommodate tropical locations, the ratio of mean monthly minimum/mean monthly maximum insolation was used to summarize insolation. Locations where the ratio of mean monthly minimum/mean monthly maximum insolation is near zero are typically near the poles and locations where the ratio of mean monthly minimum/mean monthly maximum insolation is near 1 are typically near the equator. Locations at the same latitude may have different solar insolation due to local conditions such as cloud cover, aerosols, altitude, and proximity to water.

## 4 Results

The demographics of the 5842 patients are shown in Table 1. The mean age at the time of data collection was 47.0 (14.6), and the mean age of onset was 25.2 (10.4). The GEE logistic regression model estimated the family history of mood disorders for a patient using the ratio of mean monthly minimum/mean monthly maximum insolation and gender. See Table 2, 3. The odds of a family history of mood disorders decreased as family location approached the equator and increased toward the poles. For example, if a patient and their family living in Minneapolis, MN, USA are compared to a patient and family living in Los Angeles, CA, USA, the ratio of the mean monthly minimum/mean monthly maximum insolation is 0.1567 higher in Los Angeles. This difference translates into a 12.4% ((0.211–1) * 0.1567) decrease in the odds ratio for a family history of mood disorders for the family living in Los Angeles. See Table 3. The independent variables for the model were limited to insolation at the patient's onset location and the patient's gender to minimize the possibility of overfitting a model that is estimated with imprecise data. See Table 3 for example collection locations with their ratio of mean monthly minimum/mean monthly maximum insolation values. If gender is female, the odds ratio of a family history of mood disorders increases by 21.7% compared to a male patient.

**TABLE 1:** Demographics of bipolar I patients (*N* = 5842).

Parameter	Value	*N*	%
Gender			
	Female	3364	57.6
	Male	2478	42.4
Family history of mood disorder			
	No	2847	48.7
	Yes	2995	51.3
Cohort group			
	DOB^a^ < 1940	196	3.4
	DOB ≥ 1940 and DOB < 1960	1339	22.9
	DOB ≥ 1960 and DOB < 1980	2709	46.4
	DOB ≥ 1980	1598	27.4
Parameter		Mean	SD
Age at time of data collection		47.0	14.6
Age of onset		25.2	10.4

^a^ DOB, date of birth.

**TABLE 2:** Estimated parameters explaining family history of mood disorders for patients with bipolar I disorder using ratio of mean monthly minimum/mean monthly maximum insolation and gender (*N* = 5842).^a^

Parameter				99% Wald confidence interval for exp (B)	
	df	Sig	exp (B)^b^	Lower	Upper
Intercept	1	< 0.001	1.617	1.226	2.133
Ratio mean monthly minimum/mean monthly maximum insolation	1	< 0.001	0.211	0.111	0.400
Gender = 0 (female)	1	< 0.001	1.217	1.057	1.400

^a^ Dependent variable: Family history of mood disorders (yes/no). Model: intercept, ratio of mean monthly minimum/mean monthly maximum insolation and gender (male/female).

^b^ Exp (B) is the exponentiated value of the estimated coefficient B.

**TABLE 3:** Ratio of mean monthly minimum/mean monthly maximum insolation: Example onset locations by latitude group (*N* = 5842).

Degrees latitude North + South	Onset location	Ratio of mean monthly minimum/mean monthly maximum insolation
0–9	Kampala, Uganda	0.8280
	Kuala Lumpur, Malaysia	0.8131
	Mataram, Indonesia	0.7830
	Medellín, Columbia	0.8046
	Singapore	0.8102
10–19	Bahir Dar, Ethiopia	0.7377
	Bangkok, Thailand	0.7842
	Bengaluru, India	0.7163
	Hyderabad, India	0.6793
	Mexico City, Mexico	0.7344
	Salvador, Brazil	0.6355
20–29	Hong Kong, China	0.5804
	São Paulo, Brazil	0.6079
	Taichung, Taiwan	0.4533
	Wardha, India	0.5839
30–39	Ankara, Turkey	0.2297
	Athens, Greece	0.2481
	Beer Sheva, Israel	0.3659
	Buenos Aires, Argentina	0.3105
	Cagliari, Italy	0.2510
	Cape Town, South Africa	0.3147
	Los Angeles, CA, USA	0.3585
	Melbourne, Australia	0.2600
	San Francisco, CA, USA	0.2976
	Santiago, Chile	0.2446
	Seoul, South Korea	0.3969
	Tokyo, Japan	0.4899
	Tunis, Tunisia	0.3042
40–49	Barcelona, Spain	0.2596
	Belgrade, Serbia	0.1794
	Boston, MA, USA	0.2381
	Christchurch, New Zealand	0.2303
	Grand Rapids, MI, USA	0.1866
	Halifax, Canada	0.2167
	Minneapolis, MN, USA	0.2018
	Paris, France	0.1417
	Rome, Italy	0.2211
	Siena, Italy	0.1934
	Vienna, Austria	0.1374
	Würzburg, Germany	0.1240
50–59	Aarhus, Denmark	0.0544
	Calgary, Canada	0.1368
	Dresden, Germany	0.1134
	Dublin, Ireland	0.1122
	Oslo, Norway	0.0369
	Poznan, Poland	0.0954
	Stockholm, Sweden	0.0321
	Tartu, Estonia	0.0411
60+	Helsinki, Finland	0.0299
	Khanti‐Mansiysk, Russia	0.0357
	Trondheim, Norway	0.0170

## 5 Discussion

Sunlight has widespread important impacts on the physiological and mental health of humans. Although most public education is focused upon the risks associated with excessive sunlight exposure, it is important to recognize the fundamental role of sunlight in human life and health. In addition to providing sufficient light for comfortable vision, sunlight exposure is related to vitamin D production, maintaining circadian rhythms, sleep–wake cycles, well‐being and mood, cognition, and regulation of neuroendocrine, cardiovascular, and metabolic functions [18, 19, 20, 21, 22, 23]. The effects of sunlight occur through three main routes: vision, skin absorption, and the non‐visual retinal responses to light that drive the circadian clock system and other neuronal pathways. In this analysis, we found that there was a significant relationship between a family history of any mood disorder, the ratio of the mean monthly minimum/mean monthly maximum solar insolation, and gender for patients with bipolar I disorder. This suggests a possible interaction between genetic vulnerability and environmental light exposure. In families with genetic susceptibility for bipolar disorder, the local light environment may lower the threshold for illness expression.

### 5.1 Family History

The inverse relation found between solar insolation and the family history of mood disorders suggests that family members may also be affected by solar insolation levels.

Although diverse factors may contribute to the development of bipolar disorder, multiple studies have documented the association with family history, and noted involvement of potential genetic variants [24, 25]. Children of parents who have a serious mental illness, including bipolar disorder, have an increased risk of developing a psychiatric disorder, often in childhood [26, 27, 28, 29]. Genes involved in the regulation of circadian rhythms and sleep have been linked to mental illness [30]. An early onset of BD I in childhood is associated with a family history of mood disorders and poor functional outcomes [31, 32]. Genetic variants may also contribute to disruptions in sleep and circadian rhythms found in patients with bipolar disorders [12]. Polymorphisms in some clock genes may be linked to the course of BD I disorder [33]. Patients with bipolar disorder who have a family history of suicide in first‐generation relatives are at a very high risk for suicide [25, 34]. Substance abuse and adverse childhood experiences are also risk factors for the onset of bipolar disorder [35]. Early intervention and support are often indicated for the child of a parent with bipolar disorder [29].

### 5.2 Gender Differences

Although the prevalence of bipolar disorder is similar between males and females, the presentation of symptoms and disease course may differ between the genders [36, 37, 38]. Females may have an increased risk of hypomania, rapid cycling mixed episodes, and experience mood changes across the menstrual cycle [39, 40]. Males may have an earlier age of onset of mania and bipolar disorder [41]. Females may spend a larger proportion of time with depressive symptoms [42, 43, 44]. The risk of suicide attempts is higher in females than males [45, 46, 47], but completed suicide is more common in males [48]. About 65% of individuals with bipolar disorder have one or more concurrent psychiatric disorders [49]. Males have an increased risk of simultaneous substance use and alcohol misuse disorders [49]. Females have an increased risk of anxiety and eating disorders, and of medical comorbidities including autoimmune and inflammatory disorders [44, 49, 50]. Both genders have an increased risk of cardiovascular disease which contributes to premature mortality [49, 51]. There may be gender differences in some laboratory test results in patients with bipolar disorder [52, 53]. There may be gender differences in response to treatments for bipolar disorder [37]. Women may be more concerned about side effects such as weight gain which may decrease medication adherence [54, 55]. Additionally, treatment of females for bipolar disorder must consider reproductive issues, including menstruation, childbirth and postpartum periods [36, 39, 54].

## 6 Limitations

Data collection methods were not standardized across the data collection sites. This analysis assumes that if the birth location is the same as the onset location of the patients in the database with BD I, the patient onset location is the same as the family location. A primary limitation was that family history was derived from the patient data, rather than by interviewing family members, and misclassification might bias the findings. Socioeconomic factors were not available that might influence reporting of family history. Individual variability and gender differences in light sensitivity, and the impacts of light pollution were not analyzed [56, 57, 58, 59, 60]. Light exposure patterns may differ between genders, as in the US, with females receiving less bright light than males [61]. Time of day differences in response to light exposure were not included [62]. Daily and seasonal differences in the spectral composition of light were not analyzed [63]. Seasonal patterns of patient mood variation were not available [64, 65]. The effects of living in urban, industrialized areas on light exposure were omitted [66]. The impacts of artificial light, including from occupational exposure and urbanization, may include suppression of melatonin release, interference with circadian rhythms, and adverse health effects [67, 68]. No genetic data were available. The negative effects of excessive sun exposure such as the risk of skin cancer were omitted [69]. This study did not discuss bipolar II disorder which may be more frequent in females [43, 70].

## 7 Conclusion

This study suggests that sunlight may affect the patient's family as well as the patients who have BD I with increased risk closer to the poles. Given the profound effects of sunlight on human life, health, and physiology, it is important to recognize the associations between family history, solar insolation, and gender. Psychiatrists should realize that the family of patients with mood disorders who live in the same location and experience the same solar insolation, especially females, may be at increased risk for a mood disorder.

## Author Contributions

M.B. and T.G. completed the initial draft of the manuscript, which was reviewed and approved by all authors.

## Funding

This research was supported by funds from the Medical Faculty, Dresden University of Technology, Dresden, Germany, to Michael Bauer.

## Conflicts of Interest

Lars Vedel Lessing has within the recent 3 years been a consultant for Lundbeck and Teva. Eduard Vieta has received grants and served as consultant, advisor or CME speaker for the following entities: AB‐Biotics, Abbott, AbbVie, Adamed, Adium, Alcediag, Angelini, Biogen, Beckley‐Psytech, Biohaven, Boehringer‐Ingelheim, Casen‐Recordati, Celon Pharma, Compass, Dainippon Sumitomo Pharma, Esteve, Ethypharm, Ferrer, Gedeon Richter, GH Research, Glaxo‐Smith Kline, HMNC, Intra‐Cellular therapies, Idorsia, Johnson & Johnson, Lundbeck, Luye Pharma, Medincell, Merck, Mitsubishi Tanabe Pharma, Newron, Novartis, Organon, Orion Corporation, Otsuka, Roche, Rovi, Sage, Sanofi‐Aventis, Sunovion, Takeda, Teva, and Viatris outside the submitted work. All other authors have nothing to declare.

## Data Availability

The data that support the findings of this study are available on request from the corresponding author. The data are not publicly available due to privacy or ethical restrictions.
